# Engineering of Systematic Elimination of a Targeted Chromosome in Human Cells

**DOI:** 10.1155/2017/6037159

**Published:** 2017-03-19

**Authors:** Hiroshi Sato, Hiroki Kato, Haruyoshi Yamaza, Keiji Masuda, Huong Thi Nguyen Nguyen, Thanh Thi Mai Pham, Xu Han, Yuta Hirofuji, Kazuaki Nonaka

**Affiliations:** ^1^Pediatric Oral Medicine, Division of Oral Health, Growth & Development, Faculty of Dental Science, Kyushu University, Maidashi 3-1-1, Higashi-Ku, Fukuoka 812-8582, Japan; ^2^Pediatric Dentistry & Special Needs Dentistry, Kyushu University Hospital, Kyushu University, Maidashi 3-1-1, Higashi-Ku, Fukuoka 812-8582, Japan

## Abstract

Embryonic trisomy leads to abortion or congenital genetic disorders in humans. The most common autosomal chromosome abnormalities are trisomy of chromosomes 13, 18, and 21. Although alteration of gene dosage is thought to contribute to disorders caused by extra copies of chromosomes, genes associated with specific disease phenotypes remain unclear. To generate a normal cell from a trisomic cell as a means of etiological analysis or candidate therapy for trisomy syndromes, we developed a system to eliminate a targeted chromosome from human cells. Chromosome 21 was targeted by integration of a DNA cassette in HeLa cells that harbored three copies of chromosome 21. The DNA cassette included two inverted loxP sites and a herpes simplex virus thymidine kinase (HSV-tk) gene. This system causes missegregation of chromosome 21 after expression of Cre recombinase and subsequently enables the selection of cells lacking the chromosome by culturing in a medium that includes ganciclovir (GCV). Cells harboring only two copies of chromosome 21 were efficiently induced by transfection of a Cre expression vector, indicating that this approach is useful for eliminating a targeted chromosome.

## 1. Introduction

Aneuploidy refers to an abnormal number of chromosomes, which is the hallmark of human tumors and can drive abnormal proliferation of cancer cells [1]. Defective chromosome segregation during meiosis results in gametes with an abnormality in chromosome number. Normal human cells are diploid and have 46 chromosomes arranged as 22 pairs of autosomes and one pair of sex chromosomes. Most aneuploidy in human embryos is fatal, resulting in death of the fetus before birth. However, embryos with some chromosomal trisomies survive to birth with congenital disease (e.g., trisomy of chromosome 21 results in Down syndrome). Furthermore, mosaic aneuploidy results in the failure of chromosome disjunction during cell division after fertilization. Trisomy syndromes are associated with various disorders, but it is difficult to clarify the genes responsible for them. This is because over 300 genes are present even on chromosome 21, which is the smallest human autosome. Moreover, abnormal phenotypes associated with trisomy are thought to be the result of gene-dosage imbalances. Trisomy leads to increased expression of genes encoded on the extra chromosome and affects gene expression on other chromosomes [2, 3], which makes it more difficult to understand disease etiology.

In this study, our aim was to eliminate an entire chromosome in human cells to generate normal disomic cells from trisomic cells. Previous research has indicated that XO mice lacking the Y chromosome can be created using a pair of loxP sites in an inverted orientation [4]. When male mice carrying the Y chromosome containing inverted loxP sites are mated with females carrying a Cre gene, chromosome loss is induced by recombination which is mediated by Cre present between the loxP sites on sister chromatids during embryogenesis. Moreover, targeted chromosome elimination has been achieved in mouse embryonic stem-somatic hybrid cells using a Cre-inverted loxP system that included a cassette consisting of green fluorescent protein (GFP) and drug-resistant genes bracketed by a pair of inverted loxP sites [5]. To adapt this system to human cells, we developed a modified cassette containing two inverted loxP sites, in which a counter selectable gene HSV-tk was added to efficiently select cells lacking the targeted chromosome. This cassette was integrated into the target site by homologous recombination using the clustered regularly interspaced short palindromic repeats/CRISPR-associated proteins 9 (CRISPR/Cas9) nickase system [6–8].

## 2. Materials and Methods

### 2.1. Culture of Cells

HeLa cells were cultured in Dulbecco's Modified Eagle Medium (4.5 g/l glucose) (DMEM: Nacalai Tesque, Kyoto, Japan) supplemented with 10% fetal bovine serum (FBS; Sigma-Aldrich, MO, USA), 100 units/ml penicillin, and 100 *μ*g/ml streptomycin (Life Technologies, USA) at 37°C in 5% CO_2_.

### 2.2. Preparation of Chromosome Sample and Fluorescence In Situ Hybridization

Hela cells cultured on 10 cm dishes were harvested with 0.25% Trypsin-EDTA (Gibco, NY, USA). Cells were treated with 0.075 M KCl for 40 min, fixed with ethanol-acetic acid, 3 : 1 (v/v), and placed on glass slides. For FISH analysis of chromosome 21, hybridization was performed according to the manufacturers' instructions using the chromosome 21 control probe labelled green 5-fluorescein dUTP (CHR21-10-GR, Empire Genomics, NY, USA) that specifically hybridizes to the centromeric region of chromosome 21. Following hybridization, slides were counterstained using 0.1 *μ*g/ml 4,6-diamino-2-phenylidole (DAPI, Dojindo, Japan) and mounted using ProLong diamond (Life Technologies, CA, USA). Fluorescent images were captured using Axio Imager M2 (Zeiss, Oberkochen, Germany) equipped with ApoTome2 (Zeiss, Oberkochen, Germany).

### 2.3. Plasmid Construction and Gene Targeting Mediated by CRISPR/Cas9 Nickase

To create the donor vector, cassettes consisting of marker genes NeoR, EGFP, and HSV-tk and inverted loxP sequences were constructed into the EcoRI–BamHI site of pBluescript SK. Each of the 3 kbp sequences flanking the integration site was amplified from the genomic DNA using PCR (primers: 5′-CCCCCTCGAGGTCGAAGAAATGAGCTGTCCGGCTA-3′ and 5′-TATCGATACCGTCGACAGTTTCAGTTGGACACCGA-3′ or 5′-CAGAAGCTGGGGATCTGAAATTCTGAGGCTGTTGA-3′ and 5′-TAGAACTAGTGGATCAATCACCTTCCGTCCTTCCT-3′), and they were cloned into the SalI or BamHI site of the vector plasmid by InFusion (Takara, Kyoto, Japan).

Integration of the DNA cassette into the HeLa genome was performed using the CRISPR/Cas9 nickase system. The sgRNA sequence was designed using CRISPR Direct (http://crispr.dbcls.jp/). Two annealed sgRNA oligos (sense 1: 5′-CACCGGTTGTGTCGATTAAAGTTG-3′ and antisense 1: 5′-AAACCAACTTTAATCGACACAACC-3′ and sense 2: 5′-CACCGCTTATTTCACTGTCAACGAG-3′ and antisense 2: 5′-AAACCTCGTTGACAGTGAAATAAGC-3′) were cloned into the BpiI sites of pX330A_D10A-1x2 and pX330S-2 (a kind gift from Dr. Takashi Yamamoto; Addgene #58772 and Addgene #58778), and the BsaI fragment with the sgRNA oligo in pX330S-2 was subcloned into BsaI sites of the pX330A_D10A-1x2 plasmid that expresses two sgRNAs and Cas9 (D10A) nickase [8]. CRISPR/Cas9 nickase and donor vector were cotransfected into HeLa cells using Lipofectamine 2000 (Thermo Fisher Scientific, MA, USA) according to the manufacturer's instructions. Stable clones were selected using 500 *μ*g/ml G418 (Nacalai Tesque, Kyoto, Japan), and integration was confirmed by PCR.

### 2.4. Induction of Cre Recombinase and Selection of Cells Lacking the Target Chromosome

Cells were seeded on six-well plates at a concentration of 1.5 × 10^5^ cells/well 1 day before transfection. Then, 500 ng of the Cre expression vector pCMV-Cre or pEGFP-N2 was transfected using Lipofectamine 2000 (Thermo Fisher Scientific, MA, USA), according to the manufacturer's instructions. Cells were grown for 3 days, and then they were trypsinized and replated on 10 cm dishes in selection medium with 1 *μ*g/ml GCV (Wako, Osaka, Japan). To measure the survival rate, 5 × 10^4^ cells grown for 3 days after transfection were cultured in the selection medium for 12 days. Then, colonies fixed with methanol were stained using Giemsa staining solution (Wako, Osaka, Japan), diluted by two times, for 20 min, and the colony number was counted.

### 2.5. PCR Amplification

Genomic DNA was isolated from each HeLa cell and used as a template for PCR. PCR amplification was performed using KOD FX Neo Polymerase (Toyobo, Osaka, Japan). Primer 1 and 2 sequences were 5′-GTGCATTGTTTCAAGCCACTACGTTTATGA-3′ and 5′-GGGACTGAAGTTCCATCCAA-3′, respectively.

### 2.6. Growth and Viability of Cells

Cells were seeded at a density of 5 × 10^4^ per well in a six-well plate and grown at 37°C in 5% CO_2_. The cells were harvested with 0.25% Trypsin-EDTA (Gibco, NY, USA) and mixed with an equal volume of 0.4% Trypan Blue solution (Wako, Osaka, Japan). Cell number and viability were determined using a hemocytometer. To calculate cell viability, at least 200 cells were counted in each culture after four days.

## 3. Results and Discussion

### 3.1. Preparation of HeLa Cells Integrated a Cassette into Chromosome 21

**Figure 1:**
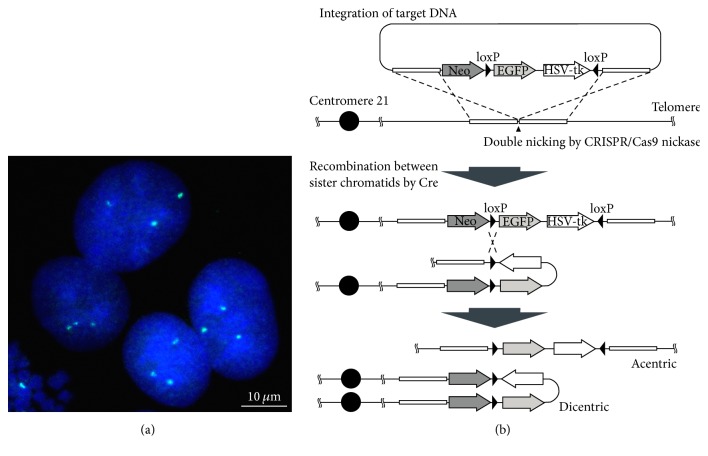
Strategy to eliminate a copy of chromosome 21 in HeLa cells. (a) Image of HeLa cell nuclei indicating three signals (green) by FISH using a chromosome 21 specific probe. DNA was stained with DAPI. (b) Scheme of recombination at inverted loxP sites by Cre recombinase. DNA cassette was integrated into chromosome 21 by homology-directed repair induced CRISPR/Cas9 nickase system. Cre-mediated recombination at the loxP sites between sister chromatids produced a dicentric and an acentric chromosome. Neo: neomycin resistance gene.

We targeted chromosome 21 for elimination in these experiments. HeLa cells that indicated three signals of chromosome 21 by fluorescence in situ hybridization (FISH) analysis were used in our approach (Figure 1(a)). HeLa cells have a hypertriploid chromosome number and normally carry three copies of chromosome 21 [9, 10]. To eliminate one copy of chromosome 21, we constructed a donor plasmid including genomic DNA fragments flanking the integration site and a DNA cassette that consisted of an enhanced GFP (EGFP) gene and HSV-tk gene bracketed by two inverted loxP sites and a neomycin resistant gene (Figure 1(b)). This donor plasmid was transfected with a CRISPR/Cas9 nickase plasmid into HeLa cells, in which homologous recombination was induced by double nicking using the CRISPR/Cas9 nickase system. Therefore, the DNA cassette integrated into an intergenic region between* RCAN1* and* CLIC6* genes on the long arm of chromosome 21.

### 3.2. Chromosome Elimination Induced by Cre Expression

**Figure 2:**
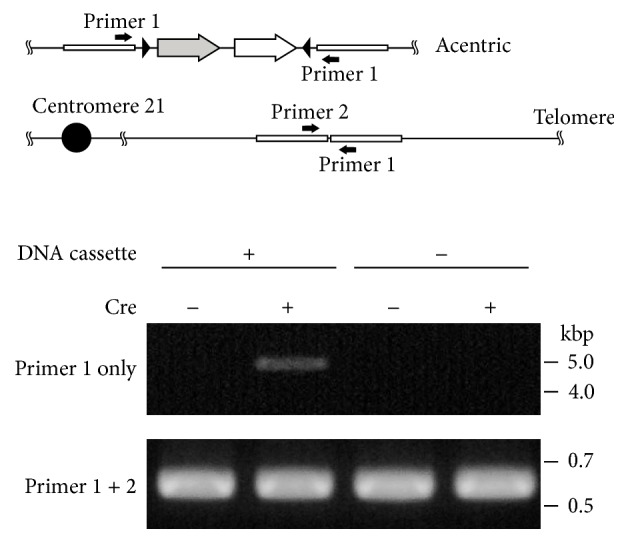
Recombination analysis between loxP sites by Cre recombinase. PCR primer-binding sites were shown on acentric chromosome and normal chromosome 21. PCR amplification was performed using genomic DNA isolated from the cells with and without integration of the DNA cassette before (Cre−) or 3 days after transfection of pCMV-Cre (Cre+).

To induce recombination between loxP sites, the Cre recombinase expression vector pCMV-Cre was transfected into the HeLa cell-integrated DNA cassette. Cre recombinase can mediate two types of recombination between loxP sites on an identical chromosome or between loxP sites on replicated sister chromatids. Recombination in an identical chromosome leads to inversion of the fragment bracketed by loxP sites. On the other hand, when loxP sites between sister chromatids are recombined, an acentric chromosome fragment and a dicentric chromosome harboring two centromeres are generated (Figure 1(b)). To confirm this recombination, we performed PCR using only one primer, which specifically detected the recombination site between sister chromatids. DNA fragments were amplified only from the genomic DNA of the cells transfected with the Cre expression vector (Figure 2). This result suggested that recombination to generate dicentric and acentric chromosomes was induced by the expression of Cre.

**Figure 3:**
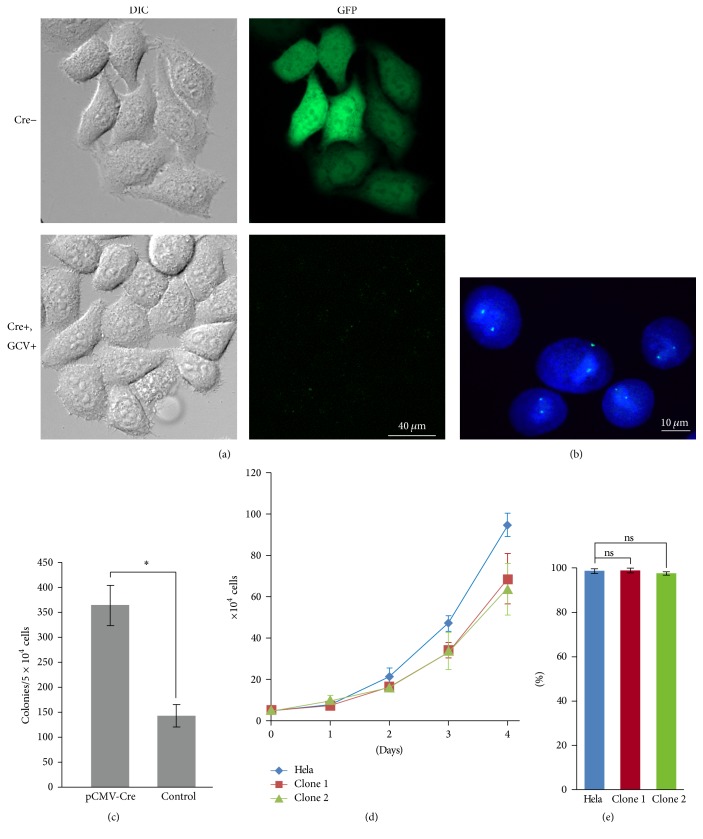
HeLa cells eliminated one copy of chromosome 21. (a) Observation of GFP expression of the cells without pCMV-Cre transfection (Cre−) or with pCMV-Cre transfection and GCV selection (Cre+, GCV+). (b) Image of the nuclei from cells selected by GCV by FISH using chromosome 21 specific probe (green). DNA was stained with DAPI. (c) Colony formation of the cells transfected with pCMV-Cre or control (pEGFP-N2). The number of colonies was estimated in the selection medium containing GCV. (d) The effect of eliminating one copy of chromosome 21 on cell growth. A HeLa cell with three copies of chromosome 21 and two clones in which a copy of chromosome 21 was eliminated, clone 1 and clone 2. These clones were isolated independently. (e) Viability of the HeLa cell and two clones. Error bars represent the standard deviation calculated from three independent experiments. Asterisks indicate significant differences (*P* < 0.005), and the ns indicates nonsignificant differences (Student's *t*-test).

These dicentric and acentric chromosomes are unstable during mitosis and would be lost from the daughter cells because of missegregation. Cells eliminating the targeted chromosome 21 could be selected by inclusion of the antiviral drug ganciclovir (GCV) in the medium. Cells including targeted chromosome 21 were killed in this selection medium because of HSV-tk genes present in the target fragment. Because of this selection, surviving cells formed colonies on the dish. EGFP signals were not detected in the selected cells but were observed in the cells before Cre expression (Figure 3(a)). Furthermore, FISH analysis indicated only two signals from chromosome 21 in the nuclei (Figure 3(b)). These results revealed that the targeted chromosome 21 was eliminated in selected cells.

Some surviving colonies were present after control plasmid transfection, indicating that the targeted chromosome 21 could be lost spontaneously from HeLa cells in the selection medium (Figure 3(c)). HeLa cells were derived from a cervical adenocarcinoma [11], and numerical chromosomal aberrations were detected by spectral karyotyping of metaphase cells [10]. An increased rate of gain or loss of whole chromosomes during each cell cycle is a common feature of many tumor cells, referred to as chromosome instability [12, 13]. Moreover, the rate of chromosome missegregation is higher in trisomic human cells than in euploid cells [14]. Therefore, extra copies of chromosome 21 integrating the DNA cassette may tend to be lost from the HeLa cells spontaneously under condition in which cells including HSV-tk gene are excluded. On the other hand, because the frequency of colony formation was significantly increased in Cre-transfected cells, targeted chromosome 21 was efficiently eliminated using our system (Figure 3(c)). Hence, this system must eliminate the targeted chromosome 21 from human cells that do not undergo spontaneous chromosome loss.

### 3.3. Alteration of HeLa Cell Features by Eliminating One Copy of Chromosome 21

To investigate the effects of the elimination of one copy of chromosome 21 in the HeLa cell, cell growth and viability were calculated (Figures 3(d) and 3(e)). The growth rate decreased in the clones in which one copy of chromosome 21 was eliminated, compared to the HeLa cell with three copies of chromosome 21. However, cell viability was unaffected by the elimination of one copy of chromosome 21. These results suggest that an additional copy of chromosome 21 may contribute to abnormal growth induction in HeLa cells.

In this study, we developed the system to eliminate the targeted chromosome 21 from human cells. Besides trisomy of chromosome 21, congenital syndromes are caused by extra copies of other chromosomes, such as trisomy of chromosomes 13 or 18, and extra copies of the X or Y chromosome. In these cases, cells with normal copy numbers of chromosomes can be generated from patient cells. Therefore, this system could be useful in the etiological analysis of chromosome abnormality phenotypes using cultured cells and for the preparation of immune tolerance sources for autotransplantation therapy.
